# Assessing Medical Ethics Knowledge and Practice Among Healthcare Professionals in Al Majma'ah, Saudi Arabia

**DOI:** 10.7759/cureus.53676

**Published:** 2024-02-05

**Authors:** Ali Faraz, Ashraf Abdelfatah Deyab, Abdulaziz Alanzan, Abdullah M Muwayni, Muteb Saeed Al Hodairy, Muteb Alharbi, Mohammad Alharbi, Sultan Alruwaili

**Affiliations:** 1 Basic Sciences, Majmaah University, Al Majma'ah, SAU; 2 Hematological Pathology and Transfusion Medicine, Majmaah University, Al Majma'ah, SAU; 3 Medicine and Surgery, Majmaah University, Al Majma'ah, SAU

**Keywords:** ksa, majmaah region, a descriptive cross-sectional study, medical ethics, attitude, knowledge

## Abstract

Background

The healthcare system is increasingly confronted with ethical challenges, necessitating a thorough exploration of healthcare providers' ethical knowledge and attitudes. This study aims to evaluate the ethical awareness and attitudes of healthcare providers in the Al Majma'ah region of the Kingdom of Saudi Arabia (KSA).

Aim

This research focuses on assessing the level of knowledge and practice regarding medical ethics among healthcare providers in a community hospital in Majmaah, KSA.

Methods

A comprehensive cross-sectional study was undertaken in the Majmaah governorate of Saudi Arabia. Data collection involved distributing a meticulously designed questionnaire to healthcare providers and faculty members affiliated with the College of Medicine. Subsequently, the acquired dataset underwent analysis utilizing SPSS software.

Results

A total of 183 participants were included in the study. The findings revealed that only 77 respondents (42.1%) considered ethical issues in their profession as extremely important. Moreover, 104 participants (56.8%) were aware of the existence of an ethics committee in their institution. A total of 113 respondents (61.7%) disagreed with the notion that ethical conduct is primarily important to avoid legal consequences, and 120 participants (65.6%) believed in the significance of in-service training on medical ethics for doctors.

The study also highlighted that 100 participants (54.6%) believed that healthcare providers' opinions supersede patient preferences. However, a majority, 163 participants (89.1%), agreed that patients should be informed of any wrongdoing, contrasting with only 20 (10.9%) who disagreed. When asked about adhering to patients' wishes despite doctors' opinions, 112 (61.2%) responded affirmatively, while 71 (38.3%) disagreed. There was also a disparity in opinions regarding the necessity of obtaining consent, with 81 (44.3%) agreeing that consent is required not only for surgical procedures but also for medications or investigations.

Furthermore, 137 participants (74.9%) recognized the importance of discussing ethical, social, and legal issues during clinical rounds alongside clinical aspects of patient care, while 46 (25.1%) disagreed.

Conclusion

Although medical ethics were introduced into the Saudi healthcare system more than a decade ago, this study underscores the ethical necessity of obtaining comprehensive informed consent prior to invasive and other medical procedures. It also highlights the significance of engaging patients in the decision-making process regarding their treatment. Well-informed patients typically exhibit higher satisfaction levels and are less inclined to pursue legal action.

## Introduction

Ethical principles, concepts, regulations, and guidelines applied to real-world medical situations play a pivotal role in ensuring that comprehensive ethical ideals are thoughtfully and judiciously applied in clinical practice [1]. The connection between clinical ethics and clinical medicine is founded on their mutual need to employ fundamental ethical principles within specific clinical situations. Medical ethics, rooted in a set of guiding principles including autonomy, non-maleficence, beneficence, and justice, serves as a reference point for healthcare practitioners when disagreements or misunderstandings arise [2]. Historical documents such as the Hippocratic Oath, dating back to the 5th century BC [3], and more recent contributions like the Nuremberg Code (1947) and the Declaration of Helsinki (1964), have significantly shaped the landscape of medical ethics [4]. Furthermore, significant events like the Deer v. Wade case in 1973 and the advancement of hemodialysis in the 1960s have contributed to the ongoing development of the ethical framework in this field [5-6]. In 2006, Saudi Arabia introduced the Patient Bill of Rights (PBR) to enhance healthcare experiences and improve the quality of care. The PBR serves as a blueprint for safeguarding patient rights, including access to suitable healthcare, respectful treatment, clear information, involvement in treatment decisions, the right to complain, and privacy protection. Patients have the autonomy to change or refuse treatments and must be informed about potential complications. The bill also mandates upfront cost information, especially in private-sector healthcare, and underscores the necessity of obtaining informed consent for all medical procedures. The PBR aims to ensure a patient-centric, respectful, and high-quality healthcare environment in Saudi Arabia [7]. Ethical principles serve as a compass for distinguishing right from wrong within a given cultural context, aligning actions with perceived moral consequences. Medical ethics specifically addresses the ethical responsibilities doctors owe to their patients, colleagues, and society as a whole. Ethics often set a higher standard of behavior than the law; what is legal is not necessarily synonymous with what is moral or ethical. Over the past few decades, undergraduate medical curricula have undergone revisions, responding to the evolving needs of the community and striving to instill a strong foundation in medical ethics among future healthcare providers. Comprehensive training in medical ethics, coupled with a favorable attitude and sufficient knowledge, equips healthcare professionals to anticipate, address, discuss, and resolve ethical dilemmas encountered in daily practice. This inclusion of medical ethics in both teaching and practice is indispensable in cultivating the competencies required to deliver ethical healthcare services [8]. This study endeavors to assess the level of medical ethics knowledge among healthcare staff, the extent to which medical ethics principles are put into practice by physicians, and the impact of such practice on the provision of appropriate patient care.

## Materials and methods

Study design

This research employed a descriptive cross-sectional study design, conducted within the College of Medicine at Majmaah University and the broader healthcare community in the Majmaah governorate, Kingdom of Saudi Arabia (KSA).

Target population

The study focused on healthcare providers within the Majmaah region's healthcare community and faculty members of Majmaah University's College of Medicine.

Inclusion criteria

The study included all active healthcare workers, irrespective of gender.

Exclusion criteria

The study excluded medical and nursing students on rotation at the hospital and studying at the College of Medicine, regardless of gender.

Sample size

The study sample consisted of healthcare providers from Majmaah hospitals and faculty members of the College of Medicine. The study employed a complete enumeration technique, ensuring data collection from the entire eligible population. No pre-determined sample size was required for this approach. A total of 189 responses were received.

Duration of the study

The study spanned a duration of six months.

Sampling technique

Data collection employed a complete enumeration technique, ensuring data was collected from the entire eligible population.

Data collection

Study participants were provided with an online Google Form to submit their responses.

Data analysis

Data collected was organized and analyzed using IBM SPSS Statistics 24. Qualitative variables were analyzed in terms of frequencies and percentages.

Ethical considerations

The study obtained ethical approval from the Majmaah Research Institutional Ethics Committee of the Basic and Health Science Research Center, Al Majmaah (IRB# MUREC-Feb.10/COM-2022/24-1). Informed consent was diligently obtained from all study subjects, and strict confidentiality measures were upheld, ensuring that all data was used solely for this research.

## Results

Table 1 presents the study's participant demographics, revealing a total of 183 individuals enrolled. Regarding the question of whether healthcare providers always know best, regardless of the patient's opinion, the responses showed that 100 participants (54.6%) agreed, while 83 (45.4%) disagreed.

**Table 1:** Knowledge of health care providers regarding medical ethics.

Questions	Agree n (%)	Disagree n (%)	Don’t know n (%)
Healthcare providers' expertise supersedes the patient's opinion.	100 (54.6%)	83 (45.4%)	N/A
During clinical rounds, it's crucial to address ethical, social, and legal aspects of patient care.	137 (74.9%)	46 (25.1%)	N/A
Patients have the right to be informed of any wrongdoing.	163 (89.1%)	20 (10.9%)	N/A
It is necessary to always inform close relatives about the patient's condition.	72 (39.3%)	85 (46.4%)	26 (14.2%)
Patient preferences should always be respected, regardless of the doctor's opinion.	112 (61.2%)	71 (38.8%)	N/A
Doctors and nurses should refuse to treat patients who behave violently.	47 (25.7%)	108 (59.0%)	28 (15.3%)
As medicine advances, confidentiality can't be maintained.	64 (35.0%)	119 (65.0%)	N/A
Consent is required not only for surgical procedures but also for medications or investigations.	81 (44.3%)	102 (55.7%)	N/A
Patients who wish to die should be assisted in doing so.	62 (33.3%)	122 (66.7%)	N/A
Doctors should ensure that their actions do not intentionally harm another, even to a small degree.	117 (63.9%)	34 (18.6%)	32 (17.5%)
Junior medical professionals tend to emulate their consultants' patient care approach.	113 (61.7%)	35 (19.1%)	25 (13.7%)
Doctors should treat patients as they would wish to be treated if they were the patients themselves.	125 (68.3%)	33 (18.0%)	25 (13.7%)
Patients who refuse treatment based on religious or other grounds should be advised to seek a doctor who aligns with their beliefs or accept the offered treatment.	84 (45.9%)	63 (34.4%)	36 (19.7%)

In the context of informing patients about wrongdoing, a significant majority of 163 participants (89.1%) expressed agreement, whereas only 20 (10.9%) disagreed. When asked if patients' wishes should always be prioritized over doctors' opinions, 112 participants (61.2%) responded affirmatively, with 71 (38.3%) holding a contrary view.

Regarding the statement, "As medicine is advancing, confidentiality can't be maintained," most participants (119 or 65.0%) disagreed, while 64 (35.0%) agreed. Concerning whether consent should be required only for surgical procedures, not for medications or investigations, more participants disagreed (120 or 65.7%) than those who agreed (63 or 34.3%).

The questionnaire also explored the topic of assisting patients who wish to die, with the majority (122 participants or 66.7%) disagreeing, while 61 (33.3%) agreed. Furthermore, 137 participants (74.9%) expressed agreement with the importance of discussing ethical, social, and legal issues alongside clinical aspects during clinical rounds, while 46 (25.1%) disagreed.

Regarding the disclosure of a patient's condition to close relatives, 72 (39.3%) agreed, 85 (46.4%) disagreed, and 26 (14.2%) responded with "Don't know." When asked whether doctors and nurses should refuse to treat violently behaving patients, 108 participants (59.0%) disagreed and would treat the patient, 47 (25.7%) agreed to refuse treatment, and 28 (15.3%) responded with "Don't know."

Furthermore, the questionnaire explored the situation where a patient refuses treatment on religious or other grounds. In response, 84 participants (45.9%) agreed that such patients should be advised to seek care elsewhere, 63 (34.4%) disagreed, and 36 (19.7%) responded with "Don't know."

Regarding the principle that doctors should avoid intentionally harming others, even to a small degree, 117 participants (63.9%) agreed, while 34 (18.6%) disagreed, and 32 (17.5%) were unsure. Finally, the majority (125 participants or 68.3%) agreed that doctors should treat patients as they would wish to be treated themselves, while 33 (18.0%) disagreed, and 25 (13.7%) responded with "Don't know."

Table 1 provides a detailed summary of these responses. We also inquired whether participants' institutions had an ethics committee. A majority of the respondents, specifically 104 individuals (56.8%), answered in the affirmative, while 34 (18.6%) responded negatively, and 45 (24.6%) indicated that they were uncertain.

Regarding the significance of ethical conduct solely to avoid legal repercussions, the majority of participants, 113 (61.7%), expressed disagreement, whereas 70 (38.3%) concurred with this perspective.

We further explored participants' views on the necessity of in-service training in medical ethics for doctors. A substantial majority, 120 participants (65.6%), acknowledged its importance, with 29 (15.8%) opposing the idea, and 34 (18.6%) remaining uncertain.

In response to the statement, "I follow the exact rules and regulations when taking a leave," a significant majority, 163 participants (89.1%), answered affirmatively, whereas only 20 (10.9%) indicated otherwise. Table 2 provides a comprehensive summary.

**Table 2:** Awareness of medical ethics among health care providers.

Questions	Agree n (%)	Disagree n (%)	Don’t know n (%)
In-service training on medical ethics is a necessity for doctors.	120 (65.6%)	29 (15.8%)	34 (18.4%)
I spend enough time explaining the purpose, nature, and consequences of treatment or procedures when obtaining informed consent from patients.	114 (62.3%)	27 (14.8%)	42 (23.0%)
Ethical conduct is important only to avoid legal action.	70 (38.3%)	113 (61.7%)	N/A
I follow the exact rules and regulations when taking leave.	163 (89.1%)	20 (10.9%)	N/A
Is there an ethics committee in your institution?	104 (56.8%)	34 (18.6%)	45 (24.6%)

Table 3 provides insights into the healthcare providers' awareness of medical ethics principles. We inquired about their recognition of specific medical ethics principles, including plagiarism, maleficence, autonomy, and fear. A majority of participants, 103 (56.3%), correctly identified autonomy as a medical ethics principle. Meanwhile, 40 (21.9%) selected maleficence, 29 (15.8%) chose plagiarism, and only 11 (6.0%) opted for fear.

**Table 3:** Health care providers' awareness of ethical principles.

Question	Plagiarism n (%)	Maleficence n (%)	Autonomy n (%)	Fear n (%)
Which of these is considered an ethical principle?	29 (15.8%)	40 (21.9%)	103 (56.3%)	11 (6.0%)

Furthermore, our survey encompassed a query regarding the perceived importance of ethical issues in their profession (Table 4). The responses were as follows: 77 (42.1%) considered it extremely important, 31 (16.9%) very important, 27 (14.8%) important, 26 (14.2%) somewhat important, and 22 (12.0%) not important at all.

**Table 4:** Participants' opinions regarding the importance of ethical issues in their profession.

Question	Not at all n (%)	Sometimes important n (%)	Important n (%)	Very important n (%)	Extremely important n (%)
How important are ethical issues in your profession?	22 (12.0%)	26 (14.2%)	27 (14.8%)	31 (16.9%)	77 (42.1%)

## Discussion

Despite the introduction of medical ethics into the Saudi healthcare system, it is evident that both patients and primary healthcare (PHC) staff have not achieved comprehensive awareness of this legislation. This implies that efforts to inform healthcare providers and recipients about ethical principles have not been effectively executed. Regarding healthcare providers' awareness of medical ethics, several noteworthy findings emerged from this study. Firstly, only 77 participants (42.1%) considered ethical issues in their profession to be extremely important. Additionally, 104 participants (56.8%) reported the existence of an ethics committee in their institutions, while the remainder either denied its presence or expressed uncertainty. Furthermore, approximately 113 participants (61.7%) disagreed with the notion that ethical conduct is primarily important to avoid legal action, indicating a broader understanding of the ethical dimensions of their profession. Additionally, 120 participants (65.6%) recognized the significance of in-service training in medical ethics for doctors. Several studies have explored the awareness of medical ethics among healthcare providers, yielding diverse results. A Saudi Arabian study investigating healthcare recipients' and providers' knowledge of the Patient Bill of Rights (PBR) found that both patients and healthcare providers exhibited a lack of essential knowledge about the PBR, emphasizing the need for increased dissemination of information tailored to the Saudi population [9]. In another study, PHC staff highlighted dissatisfaction among healthcare personnel, insufficient staffing levels, and a lack of necessary facilities as significant obstacles to implementing the Patients' Bill of Rights (PBR). These findings echo previous research in Saudi Arabia, which identified resource deficiencies, a shortage of qualified healthcare personnel, and overcrowding in PHC centers [10, 11, 12]. Addressing these issues is crucial for the successful PBR implementation, as patients may seek alternative advocacy mechanisms when their rights are not protected [13]. A 2021 study in Sri Lanka revealed a poor level of knowledge of medical ethics among doctors. Postgraduate trainees exhibited a higher level of knowledge, suggesting the need for regular in-service training on medical ethics to enhance knowledge and foster favorable attitudes and ethical conduct [14]. Similar studies in India and Pakistan reported deficiencies in medical ethics knowledge and application, emphasizing the need for improved medical ethics education in South Asian countries [14,15]. Additionally, this study found varying perceptions among participants, with a significant proportion, 100 participants (54.6%), believing that healthcare providers' opinions should prevail over patients' preferences. However, a substantial majority, i.e., 163 (89.1%), recognized the importance of informing patients about wrongdoing. The study also revealed differing views on whether patients' wishes should always supersede doctors' opinions, with 112 (61.2%) in favor and 71 (38.3%) in opposition. Furthermore, participants disagreed on whether consent should be exclusively required for surgical procedures, with 65.7% disagreeing and 34.3% agreeing. Additionally, 137 participants (74.9%) emphasized the importance of discussing ethical, social, and legal issues alongside clinical aspects during clinical rounds.

In contemporary medical practice, informed consent (IC) stands as a fundamental pillar, particularly embraced in developed nations, while remaining a contentious issue in many developing countries. The essence of IC lies in facilitating patients' awareness of the consequences of their treatment choices [16]. This significance amplifies during surgical procedures, where patients must comprehensively grasp the procedure's risks and benefits to make informed decisions. We conducted an examination encompassing aspects such as the provision of written IC forms to patients, their reading and signing of these forms, oral communication with physicians, and the potential influence of these interactions on patient decisions [16]. In a 2007 study conducted by Yousuf RM et al., it was revealed that there was widespread awareness of IC, and the "reasonable physician standard" was the preferred choice. This research delved into the examination of awareness, knowledge, and attitudes regarding IC among doctors from two distinct Asian cultures: Malaysia and Kashmir. Compared to Malaysian doctors, Kashmiri doctors displayed a tendency to exercise caution in disclosing medical information (p-value = 0.051) and were more likely to withhold it when they perceived potential harm (p-value < 0.001) or at the request of relatives (p-value < 0.023) [17]. Numerous studies emphasize the ethical imperative of providing comprehensive information before invasive procedures, underscoring the importance of involving patients in treatment decision-making. Well-informed patients tend to express higher satisfaction levels and initiate fewer legal claims. Conversely, patients who were inadequately informed about surgical risks often experienced regret following the procedure [18-20]. Recommendations for enhancing medical ethics awareness and practice include strengthening professional competencies, integrating historical examples into medical education, respecting patient autonomy while considering cultural beliefs, improving communication skills among healthcare professionals, empowering patients with essential information for informed decisions, fostering doctor-patient partnerships, promoting better listening and interactive communication skills, and advocating for more extensive studies across healthcare professions. These measures aim to bridge gaps in understanding, uphold ethical standards, and ensure patients receive the highest quality of care. The study's limitations include the reliance on self-reported knowledge and perceptions, and its sample was drawn from a single region in Saudi Arabia. The use of a self-reported questionnaire may introduce bias in the study's responses. Social desirability bias might have influenced participants' responses, as they may have been inclined to provide answers deemed favorable by others rather than expressing their genuine ethical perceptions.

## Conclusions

In conclusion, despite the introduction of medical ethics into the Saudi healthcare system, our study highlights the ongoing need for diligent information sharing before invasive medical procedures and the vital importance of involving patients in treatment decision-making. Well-informed patients tend to experience higher satisfaction levels and are less likely to initiate legal claims. This study found varying perceptions among participants regarding their awareness and perception of medical ethics. To uphold these ethical standards and ensure patient-centered care, it is imperative for physicians to continually enhance their interpersonal aptitude. This will enable them to forge strong partnerships with patients, empowering individuals to make informed choices that align with their unique values and preferences. By embracing this approach, healthcare providers can deliver more effective and patient-centric care, ultimately benefiting both patients and the healthcare system.
